
JOURNAL INFORMATION
==============================
NLM Title Abbreviation: Orthop Surg
Journal ID: Orthop Surg
NLM Journal ID: 101501666
Journal ID: OS

Orthopaedic Surgery

ISSN: 1757-7853
EISSN: 1757-7861
Publisher: Wiley

ARTICLE INFORMATION
==============================
PMCID: PMC11474633
DOI: 10.1111/os.13787
Article ID: OS13787
Article version: 1
Subjects: Issue Information, Issue Information

Issue Information

Electronic publication date: 2024 Jul 1
Publication date: 2024 Jul
Volume: 16
Issue: 7
First page: 1515
Last page: 1516
Copyright: © 2024 The Authors. Orthopaedic Surgery published by Tianjin Hospital and John Wiley & Sons Australia, Ltd.
License: This is an open access article under the terms of the http://creativecommons.org/licenses/by/4.0/ License, which permits use, distribution and reproduction in any medium, provided the original work is properly cited.
License URL: https://creativecommons.org/licenses/by/4.0/

Figure count: 0
Table count: 0
Page count: 1

source-schema-version-number: 2.0
cover-date: July 2024
details-of-publishers-convertor: Converter:WILEY_ML3GV2_TO_JATSPMC version:6.4.9 mode:remove_FC converted:15.10.2024

==============================

Volume 16 • Issue 7 • July 2024 • ISSN 1757-7861

### Orthopaedic Surgery

Open Access

REVIEW ARTICLES

1517
ARTHROSCOPIC CAPSULAR RELEASE VERSUS MANIPULATION UNDER ANESTHESIA FOR REFRACTORY FROZEN SHOULDER: A SYSTEMATIC REVIEW WITH META-ANALYSIS

1530
COMPUTERIZED VERSUS TRADITIONAL APPROACHES FOR TOTAL KNEE ARTHROPLASTY: A QUANTITATIVE ANALYSIS OF KNEE SOCIETY SCORE AND WESTERN ONTARIO AND MCMASTER UNIVERSITIES OSTEOARTHRITIS INDEX

CLINICAL ARTICLES

1538
A NOVEL LEVERAGE TECHNIQUE USING MONOAXIAL PEDICLE SCREW FOR REDUCTION OF THORACOLUMBAR COMPRESSION FRACTURES: SURGICAL TECHNIQUE AND ANALYSIS OF RADIOLOGICAL PARAMETERS

1548
MANAGEMENT OF SUSPECTED PERIPHERAL VASCULAR INJURIES IN ORTHOPEDIC TRAUMA

1555
EFFECT OF TOPICAL APPLICATION OF AN NSAID LATERAL TO THE INCISION ON POSTOPERATIVE PAIN FOLLOWING UNICOMPARTMENTAL KNEE ARTHROPLASTY: A DOUBLE-BLIND RANDOMIZED CONTROLLED TRIAL

1562
CLINICAL AND RADIOLOGICAL OUTCOMES OF CERVICAL DISC ARTHROPLASTY IN PATIENTS WITH MODIC CHANGE

1571
THE CT CLASSIFICATION OF MULTILEVEL CERVICAL OSSIFICATION OF THE POSTERIOR LONGITUDINAL LIGAMENT TO GUIDE HYBRID ANTERIOR CONTROLLABLE ANTEDISPLACEMENT AND FUSION VS. POSTERIOR LAMINOPLASTY

1581
EFFICACY AND SAFETY OF ORALLY AND INTRAVENOUSLY ADMINISTRATION OF TRANEXAMIC ACID IN PATIENTS WITH ELDERLY FEMORAL NECK FRACTURE

1592
THE COMPARISON BETWEEN SPINAL FACET JOINT TOOTHED PLATE AND TRADITIONAL PEDICLE SCREW-ROD ON REDUCTION OF THORACOLUMBAR FRACTURE

1603
TWO-LEVEL ANTERIOR CERVICAL CORPECTOMY AND FUSION VERSUS POSTERIOR OPEN-DOOR LAMINOPLASTY FOR THE TREATMENT OF CERVICAL OSSIFICATION OF POSTERIOR LONGITUDINAL LIGAMENT: A COMPARISON OF THE CLINICAL IMPACT ON THE OCCIPITO-ATLANTOAXIAL COMPLEX

1614
LESS ACETABULAR COVERAGE PREDICTS THE FAILURE OF CORE DECOMPRESSION FOR OSTEONECROSIS OF THE FEMORAL HEAD: A RETROSPECTIVE COHORT STUDY

1622
THE EFFECT OF CLAVICULAR TUNNEL POSITION ON REDUCTION LOSS IN PATIENTS WITH ACUTE ACROMIOCLAVICULAR JOINT DISLOCATIONS OPERATED WITH A SINGLE-BUNDLE SUSPENSORY LOOP DEVICE

1631
SURGICAL TREATMENT OUTCOMES OF ANTERIOR-ONLY CORRECTION AND RECONSTRUCTION FOR SEVERE CERVICAL KYPHOTIC DEFORMITY WITH NEUROFIBROMATOSIS-1: A RETROSPECTIVE STUDY WITH A 5-YEAR FOLLOW-UP

1642
3D-PRINTED PERSONALIZED POROUS ACETABULAR COMPONENT TO RECONSTRUCT EXTENSIVE ACETABULAR BONE DEFECTS IN PRIMARY HIP ARTHROPLASTY

1648
PERITENDINOUS SUBMEMBRANE ACCESS TECHNIQUE FOR MANAGEMENT OF ACUTE RUPTURES OF THE ACHILLES TENDON: A RETROSPECTIVE STUDY OF 249 CASES

1657
COMPARISON OF NOVEL 3D-PRINTED STEPPED POROUS METAL CONES AND METAPHYSEAL SLEEVES FOR RECONSTRUCTION OF SEVERE KNEE BONE DEFECTS: SHORT-TERM CLINICAL OUTCOMES

1665
SHORT-TERM MORBIDITY AND MORTALITY AFTER DISTAL FEMUR OPEN REDUCTION INTERNAL FIXATION IN THE GERIATRIC POPULATION

1673
CLINICAL EFFICACY OF INTRA-ARTICULAR TRANEXAMIC ACID INJECTION IN THE MANAGEMENT OF HEMOPHILIA WITH TOTAL HIP ARTHROPLASTY: A 24-MONTH RETROSPECTIVE COHORT STUDY

RESEARCH ARTICLES

1684
A NOVEL INTERNAL FIXATION DESIGN FOR THE TREATMENT OF AO/OTA-31A3.3 INTERTROCHANTERIC FRACTURES: FINITE ELEMENT ANALYSIS

1695
FINITE ELEMENT ANALYSIS OF SIX INTERNAL FIXATIONS IN THE TREATMENT OF PAUWELS TYPE III FEMORAL NECK FRACTURE

1710
POSTERIOR CORRECTIVE SURGERY FOR TYPE II CONGENITAL KYPHOSIS: SRS-SCHWAB GRADE 4 OSTEOTOMY OR VERTEBRAL COLUMN RESECTION?

1718
BIOMECHANICAL PROPERTIES OF NOVEL POROUS SCAFFOLD CORE AND HOLLOW LATERAL HOLE PEDICLE SCREWS: A COMPARATIVE STUDY IN BAMA PIGS

1726
RADIOLOGICAL VERIFICATION FOR THE SUITABILITY OF USING THE QUADRICEPS ANGLE TO REPRESENT ORIENTATION OF THE QUADRICEPS FEMORIS

OPERATIVE TECHNIQUE

1732
TREATMENT OF ULNAR CORONOID PROCESS FRACTURE USING THE ANTERIOR NEUROVASCULAR INTERVAL APPROACH: A RETROSPECTIVE CLINICAL STUDY WITH SHORT- TO MID-TERM FOLLOW-UP

CASE REPORT

1744
LIMITED REVISION WITH A NEWLY DESIGNED HINGE DEVICE FOR THE TREATMENT OF MEGA-PROSTHESIS HINGE FAILURE: TWO CASE REPORTS

WILEY

CHINESE ORTHOPAEDIC ASSOCIATION
中华医学会骨科学分会

View this journal online at wileyonlinelibrary.com/journal/os

### Orthopaedic Surgery

**Honorary Editor-in-Chief**
Guixing Qiu, *Beijing, China*

**Editor-in-Chief**
Xinlong Ma, *Tianjin, China*

**Associate Editors**
Cyrus Cooper, *Southampton, UK*
Benjamin G. Domb, *Des Plaines, USA*
Qing Jiang, *Nanjing, China*
Baoge Liu, *Beijing, China*
Zhuojing Luo, *Xi'an, China*
Luigi Meccariello, *Moiano, Italy*
Ling Qin, *Hong Kong, China*
Qi Yin, *Liverpool, UK*

**Editorial Board**
Elisa Belluzzi, *Padua, Italy*
Carlo Biz, *Padua, Italy*
Mary L Bouxsein, *Boston, USA*
Richard E. Buckley, *Calgary, Canada*
Zhengdong Cai, *Shanghai, China*
Wei Chai, *Beijing, China*
Bohua Chen, *Qingdao, China*
Liang Chen, *Soochow, China*
Yanxi Chen, *Shanghai, China*
Pramod Devkota, *Patan, Nepal*
Meng Fan, *Tianjin, China*
Haoyu Feng, *Taiyuan, China*
Maoqi Gong, *Beijing, China*
Yong Hai, *Beijing, China*
Wei Huang, *Chongqing, China*
Baochao Ji, *Urumqi, China*
Ayhan Kanat, *Rize, Turkey*
Kenny Kwan, *Hong Kong, China*
Sallie Lamb, *Exeter, UK*
Lifeng Lao, *Shanghai, China*
Jung-Hee Lee, *Seoul, Korea*
Guanghua Lei, *Changsha, China*
Guoan Li, *Newton, USA*
Hui Li, *Tianjin, China*
Jianmin Li, *Jinan, China*
Weishi Li, *Beijing, China*
Jianhao Lin, *Beijing, China*
Fanxiao Liu, *Munich, Germany*
Ruben Lostado, *La Rioja, Spain*
Songchen Lyu, *Harbin, China*
Weihu Ma, *Ningbo, China*
Xin Ma, *Shanghai, China*
Masaya Nakamura, *Tokyo, Japan*
Muthu Sathish, *Velayuthampalayam, India*
Wolfgang Senker, *Linz, Austria*
Sancar Serbest, *Boston, USA*
Cailiang Shen, *Hefei, China*
Bin Shen, *Chengdu, China*
Huiyong Shen, *Shenzhen, China*
Jianxiong Shen, *Beijing, China*
Puyi Sheng, *Guangzhou, China*
Junying Sun, *Soochow, China*
Shui Sun, *Jinan, China*
YasuhikoTakegami, *Nagoya, Japan*
Sakae Tanaka, *Tokyo, Japan*
Xiaodong Tang, *Beijing, China*
Fei Wang, *Shijiazhuang, China*
Weijun Wang, *Nanjing, China*
Xu Wang, *Shanghai, China*
Zheng Wang, *Beijing, China*
Jidapa Wongcharoenwatana, *Bangkok, Thailand*
Chi-Chuan Wu, *Taipei, China*
Nan Wu, *Beijing, China*
Zhihong Wu, *Beijing, China*
Yayi Xia, *Lanzhou, China*
Jianru Xiao, *Shanghai, China*
Zhongyu Xie, *Shenzhen, China*
Dan Xing, *Beijing, China*
Weiguo Xu, *Tianjin, China*
Peng Xu, *Xi 'an, China*
CaoYang, *Wuhan, China*
Pei Yang, *Xi 'an, China*
Zhaoming Ye, *Hangzhou, China*
Xiuchun Yu, *Jinan, China*
Bin Yu, *Guangzhou, China*
Yi Zeng, *Chengdu, China*
Dianying Zhang, *Beijing, China*
Hong Zhang, *Texas, USA*
Weibin Zhang, *Shanghai, China*
Xianlong Zhang, *Shanghai, China*
Jianguo Zhang, *Beijing, China*
Bin Zhao, *Taiyuan, China*
Jie Zhao, *Shanghai, China*
Yu Zhao, *Beijing, China*
Zongke Zhou, *Chengdu, China*
Yue Zhu, *Shenyang, China*
Zezhang Zhu, *Nanjing, China*

---

**Address for Editorial Correspondence:** Editorial Board of *Orthopaedic Surgery*, Tianjin Hospital, 122 Munan Road, Tianjin 300050, China (email: orthopaedicsurgery@126.com; telephone: +86 22 2833 4734; fax: +86 22 2824 1184).

**Disclaimer:** The Publisher, Tianjin Hospital and Editors cannot be held responsible for errors or any consequences arising from the use of information contained in this journal; the views and opinions expressed do not necessarily reflect those of the Publisher, Tianjin Hospital and Editors, neither does the publication of advertisements constitute any endorsement by the Publisher, Tianjin Hospital and Editors of the products advertised.

**Copyright © 2024 The Authors. Orthopaedic Surgery published by Tianjin Hospital and John Wiley & Sons Australia, Ltd**

**Copyright and Copying (in any format)**
Copyright © 2024 The Authors. Orthopaedic Surgery published by Tianjin Hospital and John Wiley & Sons Australia, Ltd. All rights reserved. No part of this publication may be reproduced, stored or transmitted in any form or by any means without the prior permission in writing from the copyright holder. Authorization to copy items for internal and personal use is granted by the copyright holder for libraries and other users registered with their local Reproduction Rights Organisation (RRO), e.g. Copyright Clearance Center (CCC), 222 Rosewood Drive, Danvers, MA 01923, USA (www.copyright.com), provided the appropriate fee is paid directly to the RRO. This consent does not extend to other kinds of copying such as copying for general distribution, for advertising or promotional purposes, for republication, for creating new collective works or for resale. Permissions for such reuse can be obtained using the RightsLink "Request Permissions" link on Wiley Online Library. Special requests should be addressed to: permissions@wiley.com

For submission instructions, subscription and all other information visit: http://wileyonlinelibrary.com/journal/os

ISSN 1757-7861 (Online)

OS.JEB.July24
